# Number and Severity of Type 2 Diabetes among Family Members Are Associated with Nutrition and Physical Activity Behaviors

**DOI:** 10.3389/fpubh.2017.00157

**Published:** 2017-07-13

**Authors:** Ann Oyare Amuta, Rahma Mkuu, Wura Jacobs, Adam E. Barry

**Affiliations:** ^1^Health Studies, Texas Woman’s University, Denton, TX, United States; ^2^Health & Kinesiology, Texas A&M University, College Station, TX, United States; ^3^Health Science, California State University Fullerton, Fullerton, CA, United States

**Keywords:** family history, type 2 diabetes, health promotion, diet, physical activity

## Abstract

**Aim:**

A binary measurement of type 2 diabetes (T2D) has been found not to influence behaviors. We aimed to examine the influence of other measures of family history such as number of relatives, genetic closeness of relatives, and severity of T2D of family members on nutrition and physical activity behaviors among college students.

**Methods:**

Students across four colleges in Texas were sampled. Multiple linear regression models, controlling for covariates, were used to model results. Cross-sectional data were used.

**Results:**

More number of relatives with T2D was associated with vegetable consumption (β = 0.131, *p* = 0.007) and exercise (β = 0.129, *p* = 0.037). Having relatives with severe T2D was associated with vegetable consumption (β = 0.157, *p* = 0.002) and exercise (β = 106, *p* = 0.027). Closer genetic relationship with someone with T2D was associated with increased vegetable consumption (β = 0.107, *p* = 0.023) and exercise (β = 0.096, *p* = 0.047).

**Conclusion:**

It is likely that the severe complications that may accompany the relatives T2D or having an immediate family member living with T2D may in fact motivate other family members without T2D to modify their attitudes, beliefs, and knowledge about T2D, thus encourage health-protective behaviors.

## Introduction

Type 2 diabetes (T2D) has been labeled an “adult onset” disease (1, 2); yet, cases of T2D continue to escalate among children and adolescents (3, 4). By year 2050, the number of adolescents with T2D will increase to approximately 84,131 (4). The growing incidence of T2D among adolescents is closely tied to the rising prevalence of obesity (5). While there are several factors that influence obesity, and subsequently contribute to T2D, lifestyle-related behaviors are chief indicators.

Physical activity (PA) and dietary behavior have been highlighted as particularly effective T2D protective factors. PA is associated with lower rates of T2D (6–9). Specifically, moderate intensity PA, such as brisk walking, significantly reduces risk of T2D (10). Among college students, aerobic PA has led to decreased risk for T2D (11). Similarly, a healthy diet significantly reduces the risk of developing T2D. For instance, nutrients and phytochemicals present in vegetables are thought to protect against the occurrence of T2D (12, 13). Leafy greens, in particular, result in improved glucose metabolism (14) and ultimately decrease risk of developing T2D (13, 15). Those who develop T2D often fail to consume five or more fruits and vegetables per day, compared to those who did not have T2D (16).

Although diet and PA play an important role in preventing the development of T2D, it is a disease involving an intricate interaction between modifiable (e.g., diet and PA) and non-modifiable risk factors (e.g., genes and family history) (17). Non-modifiable factors, such as genetics, are noteworthy given that “children and full siblings may share up to 50% of their genes, grandparents may share up to 25% of genes, and maternal or paternal cousins share 15% of genes” (18, 19). Two genes in particular, *calpain 10* and *hepatocyte nuclear factor 4 alpha*, have been identified as genes related to T2D (19). While genetic testing may not be attainable for all populations, it is noteworthy that family history might provide the necessary insights into specific conditions that “run in the family.” In addition to genetics, parents and other family members influence the development of beliefs, attitudes, and behaviors, all of which influence dietary and activity behaviors (20–22). Furthermore, family members and relatives share an environment, such as living space and physical neighborhood, which further influence T2D susceptibility (17).

Type 2 diabetes family history health education interventions have positive effects on lifestyle behaviors among adults. For example, people who know that a family member has been diagnosed with T2D are more likely to lose weight, increase PA, and practice healthy eating (23–26) as well as engage in healthier lifestyle behaviors than those who are not knowledgeable about their family history of T2D (27). That said, among college-aged adolescents, the influence of family history does not mirror the general population. Studies on normal weight and obese/overweight college students found no influence of family history status on health-protective behaviors (28–30). Some contend family history may not be as impactful among college students since they often underestimate their susceptibility to diseases (31) and do not understand the implications of family health history on an individual’s health outcomes (32). While differences noted between the general population and college students may be due to inherent differences in life stage, it is possible that the manner T2D family history has been conceptualized or operationalized is the reason for these contrasting findings. For instance, most studies have used a binary format (i.e., yes/no) to measure knowledge of T2D family history status. By employing a binary format, variability is reduced and ultimately understanding of how family history impacts T2D-related behaviors is limited. For instance, some of the intricacies that would not be captured with a binary approach include: (a) having a family member with severe T2D (i.e., lower extremity amputations), (b) how close of a genetic relationship exists between family members (i.e., first, second, or third degree relatives), and (c) the number of people in an adolescent’s family diagnosed with T2D (i.e., one person or eight people). Consequently, we sought to: (a) determine whether a binary measurement of T2D family history would exhibit a relationship to PA or nutritional behaviors among those with a positive T2D family history; (b) assess whether respondents with a family member experiencing severe T2D would engage in more frequent PA and consume greater quantities of vegetables than respondents without a family member with severe T2D; (c) quantify whether having more relatives with T2D would be associated with more frequent PA and consumption of more vegetables; and (d) whether having a closer genetic relationship with a T2D living relative would be associated with more frequent PA and consumption of greater quantities of vegetables than those who have a farther genetic relationship with a relative living with T2D.

## Materials and Methods

### Sample Selection and Participants

Sample consisted of undergraduate students (18 or older) enrolled in four colleges/universities across a southern state. Institutional size ranged from 18,413 to 58,577 students. Solicitations were sent to student’s school emails *via* administrative academic advisors. A total of 7,600 students were contacted across the four institutions, 905 of which responded (12% response rate). All study procedures were vetted by the Institutional Review Boards for each respective institution. $40 major-retailer gift card incentives were given to 13 participants *via* random drawing.

### Survey Instrument

Family history was assessed *via* the item: “Have any of your family members (mother, father, brother, sister, grandparents on both mothers and fathers side, aunts and uncles on both mothers and fathers side, and cousins on both mothers and fathers side) ever been diagnosed with T2D?” Possible responses were “No,” “Yes,” “I don’t know” and “Not applicable.” The first level of analysis replicated prior literature by utilizing the binary level of T2D family history measurement (yes/no) on health behaviors; thus, responses were coded to create a dichotomous outcome variable for knowledge of T2D family history status, such that “Yes” (coded 1) indicated a positive family history while “No,” “I don’t know,” and “Not applicable” (coded 0) indicated no family history of T2D. The second level of analyses included only respondents who had a family history of T2D and then stratified the risk by number of relatives with T2D, closeness of relationship with a relative with T2D, and severity of relatives with T2D.

#### Degree of Relationship with Family Members with T2D

Participants were asked to “select all that apply” to the following question: “which of the following relatives currently has T2D: mother, father, brother or sister, any grandparents on mothers side, any grandparents on fathers side, aunts or uncles on mothers side, aunts or uncles on fathers side, any cousins on mothers side, any cousins on fathers side.” Responses were coded to create a dichotomous outcome variable per the degree of relationship with relative with T2D. Those who reported to have a first or second degree relative living with T2D was (coded 1), while those who indicated they had only third degree relatives with T2D were (coded 0). We coded the relationship this way because one of the hypotheses that guide this paper is that having a closer genetic and most likely social/cultural/environmental relationship with a relative with T2D will heighten motivation to engage in T2D-related protective behaviors.

#### Number of Family Members with T2D

Participants were asked to write down the number of living family members with T2D.

#### Severity of Family Members’ T2D

Respondents were asked to “Please select if your family member or members with T2D has experienced or is experiencing any of the following: kidney disease, heart disease, blindness, limb amputation, or an emergency room visit for a T2D-related issue.” Possible responses were “No,” “Yes,” and “I don’t know.” Responses were coded to create a dichotomous outcome variable for T2D relative severity status, such that “Yes” (coded 1) indicated that their family member had severe T2D “No,” and “I don’t know” (coded 0) indicated that their family member did not have severe T2D.

#### Demographics

Based on the Centers for Disease Control recommendations (33), body mass index (BMI, kilograms per square meter) was calculated on self-reported weight and height. Overweight was defined as BMI 25.0–29.9, and obesity as BMI ≥30.0 (33). Other participants demographics included age (i.e., continuous variable ranging from age 18 to 27 years), biological sex (male coded 0 and female coded 1), and race/ethnicity (i.e., non-Hispanic white, African-American or black, Hispanic or Hispanic/Latino). These variables were included because there is evidence that they have associations with risk perception and health-protecting behaviors (34–37).

#### Vegetable Consumption

Vegetable consumption behaviors were measured by asking participants: “about how many cups of vegetables (including 100% pure vegetable juice) do you eat or drink each day? (1 cup of vegetables could be equal to: 3 broccoli spears, 1 cup cooked leafy greens, 2 cups lettuce or raw greens, 12 baby carrots, 1 medium potato, 1 large sweet potato, 1 large ear of corn, 1 large raw tomato, 2 large celery sticks, 1 cup of cooked beans).” Possible responses for this question were “Never” (coded as 0), “1/2 cup or less” (coded as 1), “1/2 cup to 1 cup” (coded as 2), “1–2 cups” (coded as 3), “2–3 cups” (coded as 4), “3–4 cups” (coded as 5), and “4 or more cups” (coded as 6). This question was adapted from the Health Information National Trends Survey (38).

#### PA Behavior

Physical activity was assessed *via* two items: “during the last 7 days, on how many days did you do moderate physical activities like carrying light loads, bicycling at a regular pace, or doubles tennis? Do not include walking.” 0 days (coded as 0), 1 day (coded as 1), 2 days (coded as 2), 3 days (coded as 3), 4 days (coded as 4), 5 days (coded as 5), 6 days (coded as 6), and 7 days (coded as 7); and “how much time in total did you usually spend on one of those days doing moderate physical activities?” Possible responses were open ended and participants were prompted to write the length of time in a box provided. All responses were in minutes. Responses to each of these items were multiplied to create a single, composite indicator of PA. For instance, if a respondent indicated they were at least moderately active 3 days a week, and they typically engaged in such activity for 40 min, then their weekly PA would be 120 min. “Higher scores indicated higher levels of PA each week.” This question was adapted from the Health Information National Trends Survey (38).

### Statistical Analysis

Means and SDs for continuous variables and frequencies for binary and categorical variables were calculated. Multiple linear regression was conducted to examine the impact of T2D familial risk profiles on related protective behaviors. Variables that have been established by the scientific literature as influencing health behaviors, such as age, sex, and race/ethnicity were controlled for (39–45). To analyze the binary measure of T2D family history, we used all the participants (*n* = 905); however, all other analyses of T2D family history, such as the number and type of relative with T2D, required that we extract only those with a T2D family history for the analyses (*n* = 441).

## Results

### Binary Measure of Family History and Health Behaviors

The sample was primarily female (81.5%; *n* = 736), White (58.6%; 528), and the average age was 21 (SD = 1). Almost half (48.8%; *n* = 441) had a family history of T2D. Majority of the sample consumed less than two cups of vegetables (86%; *n* = 777) (see Table 1). Based on the binary measurement of family history (yes/no) having a family history of T2D did not influence vegetable consumption (*B* = 0.135, β = 0.085, *p* = 0.115) or PA behaviors (*B* = −4.811, β = −0.004, *p* = 0.903) after controlling for demographic variables (see Table 2).

**Table 1:** Demographic characteristics of all college students (*n* = 905).

Sex	Male	163 (18.1%)
	Female	736 (81.5%)
Race/ethnicity	Black/African-American	74 (8.2%)
	White	528 (58.6%)
	Hispanic/Latino	145 (16.1%)
	Asian	41(4.5%)
	Other	115 (12.6%)
Family history of type 2 diabetes	No	462 (51.2%)
	Yes	441(48.8%)
	**Mean**	**SD**
Age	21	1
Body mass index	24	5

**Table 2:** Multiple linear regression analyses showing association between family history of type 2 diabetes (T2D) and exercise and vegetables consumption behavior’s among adolescents with a family history of T2D (*n* = 905).

	Exercise				Vegetable consumption			
Variable	*B*	SE	β	*p*	*B*	SE	β	*p*
Gender	−37.972	51.595	−0.025	0.462	0.009	0.112	0.003	0.934
Age	16.616	14.751	0.039	0.260	0.099	0.032	0.106	0.002
Hispanic_Latino	57.255	56.490	0.042	0.311	−0.079	0.123	−0.027	0.520
Black_African_American	20.918	83.437	0.011	0.802	−0.332	0.181	−0.076	0.067
White	35.949	57.870	0.029	0.535	0.215	0.126	0.078	0.087
Body mass index	−7.378	3.663	−0.069	0.044	−0.009	0.008	−0.039	0.245
Family history binary	−4.811	39.322	−0.004	0.903	0.135	0.085	0.053	0.115
	*R*	0.09			*R*	0.20		
	*R*^2^	0.01			*R*^2^	0.03		

### A More Nuanced Approach to Measuring Family History and Health Behaviors

Of the total number of participants (*n* = 905), approximately 441 reported a family member with T2D. The sample was primarily female (85.5%; *n* = 375). Most respondents were white (60%; *n* = 265). The average age of [Sec S2-1]participants was 20 years old (SD = 1). About half of the participants had a family member with severe T2D (47.2%; *n* = 207), and majority had a first or second degree relative diagnosed with T2D (76.4%; *n* = 337). Number of relatives with T2D ranged from 1 to 9 (mean score = 2.0; SD = 1.3). Mean number of cups of vegetables eaten in a week was 2.23 (SD = 1.3), and average number of minutes PA per week was 75.7 (SD = 113.5). Mean BMI was 25.3 (SD = 5.8) (see Table 3).

**Table 3:** Demographic characteristics of college students with a family history of type 2 diabetes (T2D) (*n* = 441).

Sex	Male	64 (14.5%)
	Female	375 (85.5%)
Race/ethnicity	Black/African-American	50 (11.3%)
	White	265 (60%)
	Hispanic	126 (28.6%)
Genetic closeness to relative with T2D	First and second degree	337 (76.4%)
	Third degree	104 (23.6%)
Severity of relatives T2D	Yes	207 (47.2%)
	No	231 (52.7%)
	**Mean**	**SD**
Number of relatives with T2D	2.0	1.3
Age	20	1.4
Body mass index	25.3	5.8
Physical activity	75.7	113.5
Vegetable consumption	2.2	1.3

Increased number of relatives with T2D was significantly associated with PA (*B* = 27.4, β = 0.11, *p* = 0.037) and with vegetable consumption (*B* = 0.129, β = 0.131, *p* = 0.007) after controlling for demographic variables (see Table 4). Having a closer genetic relationship with relatives with T2D was significantly associated with PA (*B* = 83.4, β = 0.096, *p* = 0.023); and with vegetable consumption (*B* = 0.362, β = 0.107, *p* = 0.023) after controlling for demographic variables (see Table 5). Having a relative with severe T2D was significantly associated with PA (*B* = 73.5, β = 0.106, *p* = 0.002), and with vegetable consumption (*B* = 0.501, β = 0.157, *p* = 0.002) after controlling for demographic variables (see Table 6).

**Table 4:** Multiple linear regression analyses showing association between number of relatives with type 2 diabetes (T2D) and exercise and vegetables consumption behavior’s among adolescents with a family history of T2D (*n* = 441).

	Exercise				Vegetable consumption			
Variable	*B*	SE	β	*p*	*B*	SE	β	*p*
Gender	−42.682	47.494	−0.043	0.369	−0.292	0.174	−0.079	0.094
Age	19.233	12.497	0.076	0.125	0.116	0.046	0.121	0.012
Hispanic_Latino	26.518	47.571	0.035	0.578	−0.265	0.174	−0.092	0.129
Black_African_American	−65.525	67.722	−0.059	0.334	−0.443	0.248	−0.106	0.075
White	−19.457	48.538	−0.027	0.689	0.268	0.178	0.099	0.132
Body mass index	−5.603	2.900	−0.094	0.054	−0.021	0.011	−0.094	0.049
Number of relatives with T2D	27.427	13.094	0.105	0.037	0.129	0.048	0.131	0.007
	*R*	0.18			*R*	0.29		
	*R*^2^	0.03			*R*^2^	0.09		

**Table 5:** Multiple linear regression analyses showing association between genetic closeness with relatives with type 2 diabetes (T2D) and Exercise and vegetables consumption behavior’s among adolescents with a family history of T2D (*n* = 441).

	Exercise				Vegetable consumption			
Variable	*B*	SE	β	*p*	*B*	SE	β	*p*
Gender	−80.038	50.291	−0.076	0.112	−0.215	0.191	−0.053	0.259
Age	5.579	13.222	0.021	0.673	0.097	0.050	0.093	0.054
Hispanic_Latino	93.421	49.949	0.115	0.062	−0.199	0.189	−0.063	0.294
Black_African_American	−41.729	71.418	−0.035	0.559	−0.351	0.271	−0.077	0.196
White	34.690	51.199	0.046	0.498	0.326	0.194	0.110	0.094
Body mass index	−4.234	3.076	−0.067	0.169	−0.025	0.012	−0.104	0.029
Genetic closeness to relative with T2D	83.440	41.949	0.096	0.047	0.362	0.159	0.107	0.023
	*R*	0.19			*R*	0.29		
	*R*^2^	0.04			*R*^2^	0.10		

**Table 6:** Multiple linear regression analyses showing association between severity of relatives type 2 diabetes (T2D) and exercise and vegetables consumption behavior’s among adolescents with a family history of T2D (*n* = 441).

	Exercise				Vegetable consumption			
Variable	*B*	SE	β	*p*	*B*	SE	β	*p*
Gender	−45.999	47.249	−0.047	0.331	−0.483	0.212	−0.107	0.91
Age	19.775	12.445	0.078	0.113	0.038	0.056	0.033	0.023
Hispanic_Latino	31.698	48.198	0.041	0.511	0.345	0.216	0.098	0.493
Black_African_American	−73.787	68.168	−0.066	0.280	−0.101	0.305	−0.020	0.111
White	−27.977	48.685	−0.039	0.566	−0.123	0.218	−0.037	0.741
Body mass index	−5.681	2.870	−0.096	0.048	0.040	0.013	0.146	0.574
Severity of relatives T2D	73.550	33.225	0.106	0.027	0.501	0.149	0.157	0.002
	*R*	0.20			*R*	0.30		
	*R*^2^	0.39			*R*^2^	0.89		

## Discussion

Results suggest that there is an association between practicing health-protective behaviors and familial risk profile. In particular, having (a) relatives with severe T2D, (b) greater number of relatives with T2D, and (c) a close genetic relationship with a relative with T2D is associated with increased PA levels and consumption of healthy food options. It is noteworthy that these associations would not have been detectable unless a more nuanced assessment of T2D risk was employed. In other words, a dichotomous family history measure, which simply indicates whether or not a family member has been diagnosed with T2D, did not identify a statistically significant relationship between T2D family history and health behaviors. Consequently, we recommend practitioners and researchers cease using simple, dichotomous measures of family history, which ignore important factors, such as familial risk profiles and “closeness” of relatives. If dichotomous measures are employed, it is imperative that follow-up assessment examine contextual factors (i.e., risk, “closeness”) for those indicated presence of a family T2D history. While the results herein document associations between T2D risk and PA and dietary behaviors, it is beyond the scope of this investigation to outline the underlying mechanisms of these associations. Previous research, however, contend that representations of illness can create feelings of vulnerability, which can in turn motivate an individual to action (46). For example, if an individual observes a close relative constantly battling complications associated with T2D (e.g., emergency department visits, blindness, limb amputation), their specific illness representation will be quite different than a person who has never observed, firsthand, the impact of T2D on a family member. These unique individual experiences, in turn, likely influence an individual’s perceived risk of T2D and feelings of vulnerability to the disease. Experiencing T2D in the family, particularly when the illness has become severe or when several relatives are diagnosed with T2D, can heighten emotions. Emotions, according to Ferrer et al. (47), are defined as “a relatively brief affective reaction to a specific person, situation, or sensory stimuli” (47). Emotional feelings have been found to strongly influence health decision-making and have demonstrated very strong influences on engaging in health behaviors (47–50). Moreover, similarities and/or dissimilarities between an individual and their respective family member living with T2D (e.g., biological sex, age, personality type, lifestyle behaviors, physical stature, and body features) may also be influential factors on perceived risk and feelings of vulnerability to T2D. Future examinations should investigate the impact of these factors, and whether they mediate the relationship between T2D and engagement in protective behaviors, such as PA and dietary behaviors.

Each of the individual components of the familial risk profile (i.e., number of family members with T2D, degree of relationship to relative with T2D, and severity of relative’s T2D) was an indicator of risk for T2D and demonstrated a statistically significant relationship with preventive health behaviors. It is possible that this relationship is because family members encourage healthy behaviors among the youth and adolescents in the family to protect them from developing T2D, particularly when the family is burdened with disability and financial stress. Although friends are the most influential on adolescent PA behaviors (29, 30), it is likely the severe complications that may accompany a family members’ T2D may, in fact, motivate other family members without T2D to modify their attitudes, beliefs, and knowledge about T2D, thus encourage health-protective behaviors. Future investigations should seek to explore the relationships among the aforementioned constellation of factors.

### Implications and Limitations

Despite the novel nature of this study, there are several limitations, which need to be noted. First, the data are cross-sectional, which limits inferring causality. Second, it is possible that cases of T2D are still undiagnosed; thus, participants may have a family history of T2D but were unaware (51). Additionally, all data for this study were self-report. Women were also overrepresented in our sample, which may have biased our results. The *R*-squared values in the results are low; this could be because some confounding variables were not included in the analyses. For example, the amount of time an individual has known about their family member’s T2D may influence when participation in T2D-related behaviors begin. Suppose most of our respondents knew about their family members T2D for only 1–3 weeks before participating in this study, their responses may have influenced the outcomes and strength of the *R*-squared values. Finally, the survey questions examining PA specifically excluded walking, which may have resulted in an underestimate of PA behaviors.

Several notable implications stem from this study. Although it has become increasingly popular to collect information of T2D family history, most of the information collected is in a binary form (i.e., “do you have a family member living with T2D?” Yes or No). As the results herein highlight, more nuanced family history measures represent better measurement tools. Thus, collecting information on various familial risk profiles such as severity, type, and number of relatives with T2D can provide greater insights into an individual’s level of risk, particularly among overweight/obese populations. Stratifying familial risk information can also provide avenues to systematically organize screening procedures. Early screening among those ranking high in the strata is essential because the time lag between the onset of T2D and diagnosis is approximately 7 years (52). These high-risk family members can be easily identified based on their familial risk profile for opportunities for both primary and early secondary prevention as a significant number of people diagnosed with T2D already have complications at the time of diagnosis (52–54). Similarly, the period when family members experience a relative’s T2D severity may create an opportunity to educate them about the important relationships of health-protective behaviors, T2D, and overall health status to protect them from developing T2D. We provide insight into possible mechanisms to improve the health-related behavior of adolescents with a family history of T2D (particularly those with several family members with T2D, those who have parents and siblings living with T2D, and those who have a relative with severe T2D).

## Ethics Statement

All study procedures were vetted and permitted by the Institutional Review Boards of Texas A&M University, Prairie View A&M University, and University of the Incarnate Word. Since it was an online and completely anonymous survey, participants were given informed consent to read via email solicitations sent out, all participants were advised that clicking on the survey link indicated consent.

## Author Contributions

AA came up with the idea for the paper, ran all the analyses, and drafted the methods and results. WJ drafted the introduction. RM drafted the discussion. AB contributed throughout the paper and edited the paper.

## Conflict of Interest Statement

The authors declare that the research was conducted in the absence of any commercial or financial relationships that could be construed as a potential conflict of interest.
